# Variable Effects of Apormorphine

**Published:** 1908-08

**Authors:** Frank K. Boland

**Affiliations:** Atlanta, Ga.


### VARIABLE EFFECTS OF APOMORPHINE.

FRANK K. BOLAND, M. D., ATLANTA, GA.

Apomorphine is one of the most active and useful drugs in the materia medica, and though the ultimate effect of its administration is generally uniform, the primary effect, in my experience, is subject to considerable variation. The ultimate effect is to produce relaxation, with or without sleep. Accompanied by increased pulse rate and sweating, this may be the only effect. Usually, however, it is preceded by slight nausea and profuse vomiting, though in two of my cases it was preceded by convulsions, where none had existed before its administration.

These differences in action may be due to idiosyncrasy in the patient or chemical variation in the drug. I have seen distinctly opposite effects occur in patients, apparently suffering in the same way from the same trouble, to whom the same dose was given, and have seen a typical result, if there is such, occur in a patient to whom a tablet a year old, and black and shrunken from exposure, was given.

Apomorphine is the hydrochoride of an artificial alkaloid prepared from morphine of codeine. It is not an old preparation, and has been in common use in this section of the country for not over twenty years. The drug is recommended as an emetic, an expectorant, and a motor sedative. Dr. J. S. Horsley, of West Point, Georgia, in the Medical Record, December 6, 1890, was one of the first to call attention to its use for the last named purpose, and reported a successful cure of strychnine poisoning from its administration, without the production of emesis. Dr. Horsley since advocates apomorphine for the control of all spasms not caused by lesion of the brain or spinal cord. The drug is given hypodermically, and I do not believe it is very generally employed as an expectorant.

My observation of the effect of apomorphine, besides showing its variability, leads to the conclusion that the doses usually recommended in the text-books are too large. My dose for an adult is 1-20 grain. This will usually produce the emetic and relaxing result desired, and if it does not, it can be repeated. A dose of 1-15 grain should be the maximum, and given then only in desperate cases of poisoning. The drug should be used only as a last resort in children.

I have never seen a death from the administration of apomorphine, but have had one case in which death seemed imminent from the use of 1-15 grain, and saw another with a colleague in which 1-10 nearly caused a fatal result. The patients in both instances were healthy, robust men. Dr. Craig Barrow, of Savannah, saw a case in which 1-12 grain caused death in a nine year old child suffering from tubercular peritonitis.

The following cases are typical of the points I wish to emphasize:

Case I. Very frail man, aged 35, steady drinker. Condition now bordering on delirium tremens, very nervous and talkative. Large doses of ordinary sedatives of no effect. 1-15 grain apomorphine, hypodermically, caused patient to go to sleep in fifteen minutes without nausea or vomiting. Pulse was slightly accelerated, and patient was in mild sweat.

Case II. Irish stone cutter, powerful, plethoric man, who got on occasional mean sprees. 1-15 grain apomorphine caused him to vomit copiously within five minutes, and break out in profuse sweat. In the midst of this, he fell over on the floor, limp as a rag, respiration very shallow, and practically without pulse. Within fifteen minutes after giving 1-30 grain strychnine, pulse and respiration had returned to normal, and he dropped off into a quiet sleep. Ever since this case, as soon as I give apomorphine, I always load my syringe with strychnine.

Case III. Married woman, aged 22. Suffering from severe epigastric pain for twelve hours, without nausea, tenderness, or rise of temperature. All other emetics proving unavailable, I gave 1-25 apomorphine, and within ten minutes she vomited a card-party meal which she identified as having taken twenty-four hours before. Afterwards she was entirely relieved of her pain, and went to sleep.

Case IV. This was a remarkable case. The patient was a very delicate man, aged 30, who had been affected with alcoholic neuritis for several days, complicated by retention of urine, for which catheterization was necessary. He was also suffering from alcoholic gastritis, and able to retain but little on his stomach. Suddenly he developed an acute and violent delirium, and it became necessary to restrain him by force. All other sedatives, including morphine, affording no relief, I was anxious to try apomorphine, but, having just seen case II, was dubious about using it on a man in such a weak condition. Upon the advice of a consultant, however, I administered 1-15 grain. For about ten minutes this quieted the patient, without causing nausea or vomiting, and then he suddenly had a distinct convulsion, with marked opisthotonos. This continued for four or five minutes, then stopped, after which he had two similar attacks of less violence. Finally, he too went to sleep, and though sick for several weeks longer, he required no more apomorphine, and eventually recovered.

It will be seen from these cases that though the ultimate effect of the drug is constant, there are several different routes of reaching it. My conclusions in regard to apomorphine are that it is the most valuable emetic we possess, and one of the most valuable antispasmodics, but that it is a dangerous remedy, and must be used with the utmost caution. I trust the publication of these experiences will be the means of bringing out the experiences of others concerning a drug about which there yet seems something to be learned.

821 Century Building.

